# Substance Misuse Pattern and Sexual Risk Behavior among Street Adolescents in Central Java, Indonesia

**DOI:** 10.34172/jrhs.11317

**Published:** 2025-09-15

**Authors:** Zahroh Shaluhiyah, Bagoes Widjanarko, Syamsulhuda Budi Mustofa, Priyadi Nugraha

**Affiliations:** ^1^Department of Health Promotion and Behavioral Sciences, Faculty of Public Health, Diponegoro University, Semarang, Indonesia

**Keywords:** Street youth, Sexual behavior, Substance use, Alcohol, Smoking, Pornography

## Abstract

**Background:**

Substance use is common among street adolescents and is strongly associated with sexual risk behavior as well as vulnerability to sexually transmitted infections. Therefore, this study was conducted to explore patterns of substance use and the association with sexual risk behavior among street adolescents in Central Java, Indonesia.

**Study Design:**

A cross-sectional study.

**Methods:**

In this cross-sectional study, data were collected from 248 street adolescents through face-to-face interviews using a validated questionnaire. Data analysis was conducted using descriptive statistics, chi-square tests, and multivariate logistic regression in SPSS version 25.0.

**Results:**

More than a quarter of adolescents reported engaging in high-risk sexual behavior. Heavy smoking, alcohol use, and frequent exposure to pornography were prevalent. Alcohol use was significantly associated with sexual risk behavior, with adjusted odds ratios ranging from 3.26 to 4.38 across Models I–III. Furthermore, frequent exposure to pornography showed a strong association, with odds ratios of 3.02 (Model I) and 4.20 (Model II). These associations remained significant after adjusting for demographic and behavioral variables.

**Conclusion:**

Substance use, particularly alcohol consumption, and frequent exposure to pornography were significantly associated with sexual risk behavior among street adolescents. Therefore, interventions should be developed to address the specific needs of this population. Adolescents engaged in high-risk behavior, such as substance use and sexual risk behavior, required high attention and specific treatment options.

## Background

 According to UNICEF, street adolescents are children under 18 who have abandoned families and immediate surroundings to live on street.^1,2^ These children can be found in both developing and developed countries, with poverty being primarily responsible for selecting street life.^3^ Globally, the estimated number of street adolescents is approximately 150 million, but this figure is debated and the exact number is unknown.^1,3^

 UNICEF categorizes street adolescents into 3 groups. These groups included the homeless living and sleeping on the street, adolescents working but returning home at night to their families, and those at risk.^4-6^ Street adolescents face numerous challenges, including physical and psychological exploitation, social abuse, substance misuse, addiction, HIV infection, as well as a lack of education and health services.^7,8^ Substance use, including alcohol and drugs, can lead to physical health problems, cognitive and social disorders, as well as sexual risk behavior.^9^

 According to the Indonesian Institute of Sciences and the Indonesian National Narcotics Board, 5 out of 100 Indonesian adolescents aged 15 to 18 are reported to have serious drug use problems. In 2018, the Indonesian Basic Health Research found that the prevalence of smoking among adolescents aged 10–15 and 15–19 years was 0.7% and 12.7%, respectively. Additionally, 3.8% of adolescents consumed alcohol for the first time between 5 and 9 years old.^10,11^ These data emphasize that substance use is a pressing public health concern in Indonesia and has a tendency to intersect with other risk behaviors among the vulnerable population.^12-14^

 Central Java is among the Indonesian provinces with a high and increasing number of street adolescents, due to persistent economic hardship caused by the COVID-19 pandemic. In 2021, the Ministry of Social Services recorded over 4500 street children in the region.^15^ These adolescents are frequently exposed to various health risk behaviors, including smoking, alcohol consumption, use of bootleg drinks and drugs, as well as early exposure to pornography. Sexual risk behaviors such as bartered or transactional sex, multiple and changing partners, as well as experiences of violence, are commonly reported, particularly in Semarang City, often rooted in neglectful or permissive family environments.^6^ Previous studies reported early sexual debut at age 11, where unprotected intercourse and high partner turnover put adolescents at elevated risk of STIs, HIV, unintended pregnancies, and abortion.^16-21^ Motivations for sexual activity range from financial need and peer pressure to substance use and coercion.^17^

 Drug addiction has been related to high-risk sexual practices, including unprotected sex, multiple and same-sex partners, and transactional encounters.^22^ Despite these concerning trends, there are limited empirical studies investigating the co-occurrence and interrelation of risk behavior among street adolescents. Therefore, this study was conducted to describe the patterns of substance use and sexual risk behavior among street adolescents. The analysis was conducted to examine the associations between substance misuse and sexual risk-taking within the high-risk group in Central Java. By exploring both the extent of this behavior and the interrelationship, this study contributes to the limited literature on street adolescents in Indonesia, providing valuable information for policy-making and intervention design.

## Materials and Methods

###  Study Design 

 A cross-sectional design was used in this study to assess the patterns and relationships between substance use and sexual risk behavior among street adolescents in Central Java, Indonesia. Data were collected at a single point in time to provide a snapshot of the behavior prevalence.

###  Participants and Sample Size

 The quantitative analysis was conducted in Semarang city and the surroundings, covering 404 km west of Jakarta, which comprised 248 street adolescents aged 10 to 18. There were approximately 4602 street children aged from 5 to 17 years old registered with a local non-governmental organization (NGO) or foundation in Central Java Province. The respondents of this study were street adolescents who have been living in Central Java, specifically Semarang, Pekalongan, Batang, and Pemalang Regencies, registered as a member of a local NGO and were willing to participate. Semarang had 384 street children, including adolescents who were registered in some foundations, while Pekalongan, Pemalang, and Batang had 100, 65, and 35 street children, respectively.

**Figure 1:**
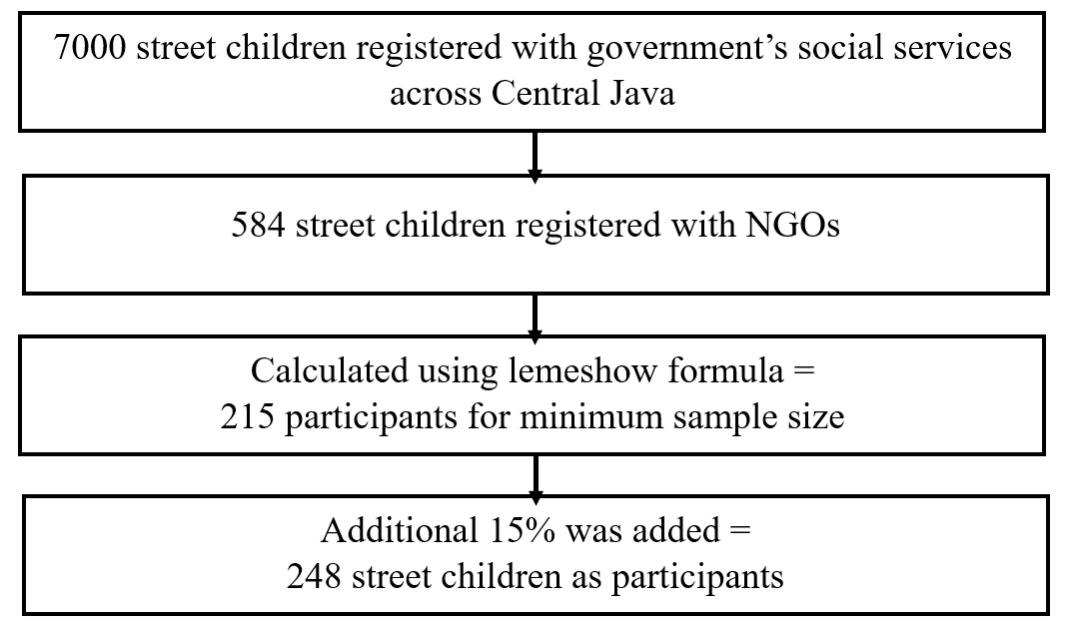
Sample and participants

 The sample size was calculated using the Lemeshow formula, with a confidence level of 95%, a proportion (p) of 0.3 based on previous results regarding the prevalence of high-risk behavior among street adolescents,^23,24^ and q = 1−p. A precision level (d) of 0.1 was applied, leading to a minimum required sample size of 215. To accommodate potential non-responses, an additional 15% was added, obtaining a final sample size of 248 respondents (Figure 1).

###  Procedures and Data Collection

 A purposive sampling strategy was used, and eligible street adolescents were identified through local NGOs experienced in youth outreach. These NGOs helped locate respondents who met the inclusion criteria. In Semarang, 5 NGOs (Yayasan Emas Indonesia (YEI) Kota Semarang, YEI Pondok Boro, Rumpun Bangjo, Anantaka, and Komunitas Harapan) provided access to registered street adolescents. In other regencies, data were obtained from local social welfare offices. The use of purposive sampling through NGOs and geographically focusing on Semarang and surrounding regencies showed both ethical and logistical realities in working with the vulnerable population. This approach was selected for its practical feasibility and because registered street adolescents are often not physically present at their listed locations due to high mobility.

 To reach this mobile population, respondents were selected directly from shelters, ensuring that they met the criteria for street children and simplifying outreach. From the available lists, adolescents aged 10–18 were identified, and a minimum sample size was calculated. Convenience sampling was applied to recruit available and willing respondents. Interviews were conducted when adolescents returned to the shelter. For those with parents, consent was obtained and some parents requested additional incentives. For adolescents without parents, permission was obtained from NGOs or social welfare caregivers.

 Data collection was carried out from March to October 2021, using a questionnaire covering demographics, knowledge and attitudes toward sexuality, peer and parental influences, substance use, and sexual risk behavior. This questionnaire was administered in Javanese and Indonesian to ensure comprehension. Face-to-face interviews allowed for clarification when needed and were conducted by 6 master’s students in Public Health Promotion, all with professional health backgrounds.

 In Central Java and across the island, street adolescents tended to share core sociocultural and behavioral characteristics due to the nature of the street lifestyle. The subculture of street adolescents in urban and peri-urban Java is marked by common experiences. These experiences included family disruption, school dropout, economic survival through informal work (scavenging, busking, etc), and reliance on peer networks for emotional and material support. The peer groups often function as surrogate families and exert a strong influence on norms surrounding masculinity, risk-taking, substance use, and sexual behavior. Risk behavior is not only normalized but also serves as a mechanism of identity formation, resilience, and group belonging. Since these conditions are not unique to Semarang or the selected regencies and are also prevalent in other cities across Java, behavior patterns and vulnerabilities observed in the sample are likely to be broadly representative of street adolescents in similar urban settings throughout the region. Therefore, the results of this study can considerably inform regional-level interventions and serve as a foundation for more geographically diverse investigations in the future.

 Informed consent was obtained from all participants prior to data collection. For minors accompanied by parents, parental consent was secured, with a few parents requesting additional incentives. For unaccompanied minors, permission was granted by designated NGO or social welfare caregivers in accordance with established ethical protocols. This study was approved by the Ethics Committee of the Faculty of Public Health, University of Diponegoro (192/EA/KEPK-FKM/2022).

###  Instruments and Measures

 The questionnaire was carefully developed and pilot-tested with a subset of the target population to assess clarity, relevance, validity, and reliability. The pilot study showed good internal consistency, with a Cronbach’s alpha of 0.81, indicating high reliability. Sexual behavior was the primary dependent variable in this study, categorized as high-risk and low-risk. Adolescents who had experienced sexual intercourse were automatically classified as engaging in high-risk sex behavior. However, those who had not experienced sexual intercourse were categorized as engaging in low-risk sex behavior. This binary classification aligns with previous research involving adolescents in vulnerable settings, where sexual initiation is often associated with increased health risks due to limited access to health education, low condom use, and peer-driven normalization of unsafe practices.^25^

 Smoking habits were classified as light or heavy, with heavy smoking defined as smoking more than one pack per day (over 7 packs per week).^26-30^ Alcohol use, including beer, wine, traditional drinks, and mixtures with drugs, was categorized as ever or never.^29,30^ Drug use was similarly classified and included substances such as dextromethorphan, methamphetamine, mixed pills, marijuana, heroin, ecstasy, and gasoline. Exposure to pornography was measured as a binary variable (exposed or not exposed), based on lifetime experience. Alcohol consumption was also recorded as ever or never used.^31,32^

 Covariates included gender (male/female) and age (< 14 or ≥ 14 years) based on WHO adolescents risk classifications, and occupational status (working or not working). Knowledge of sexuality was assessed through a 17-item questionnaire, attitudes toward sexuality with 14 items, and friends’ sexual behavior using a 10-item scale. Responses were categorized as high-risk or low-risk based on standard deviation cut-off values.

###  Data Analysis 

 Data were checked for consistency, coded, cleaned, and analyzed using SPSS version 25. Descriptive statistics (frequencies, percentages, mean, and SD) were used to summarize demographic and independent variables. The chi-square test was used to examine the relationship between substance use and sexual risk behavior, with significance set at *P* < 0.05. To explore associations with dependent variables, bivariate and multivariate logistic regressions were applied. A total of 3 models were built using a significance threshold of *P* < 0.10 for variable inclusion and 95% CI with *P* < 0.05 considered significant. The *P* < 0.10 threshold was selected following the recommendation of Hosmer and Lemeshow, who suggested using a more inclusive criterion during variable selection in exploratory public health. Model I included all variables (demographic, behavioral, and attitudinal) to provide a broad overview. Model II focused on substance use and exposure to pornography, while Model III assessed substance use only. This method allowed for a clearer understanding of general and specific predictors of sexual risk behavior, showing how each set of variables influenced the results.

## Results

###  Characteristics of Respondents

 A total of 248 street adolescents participated in this study, with a mean age of 14.69 ± 2.823 years. The majority of respondents (62.1%) aged between 10 and 14 years, while the remaining 37.9% aged 15 to 18 years. In terms of gender distribution, male adolescents accounted for a higher proportion (60.9%) compared to female adolescents (39.1%).

 Regarding occupational status, a significant number of respondents (42.3%) reported having no job. Among those who were employed, the most common type of work was begging (33.9%), followed by informal and irregular work (23.8%), such as busking, parking assistance, or selling goods. A small proportion engaged in specific tasks such as newspaper vending (0.6%), scavenging (1.2%), or working as shopkeepers, florists, or food vendors (20.5%).

###  Knowledge, Attitudes, and Behavior 

**Figure 2:**
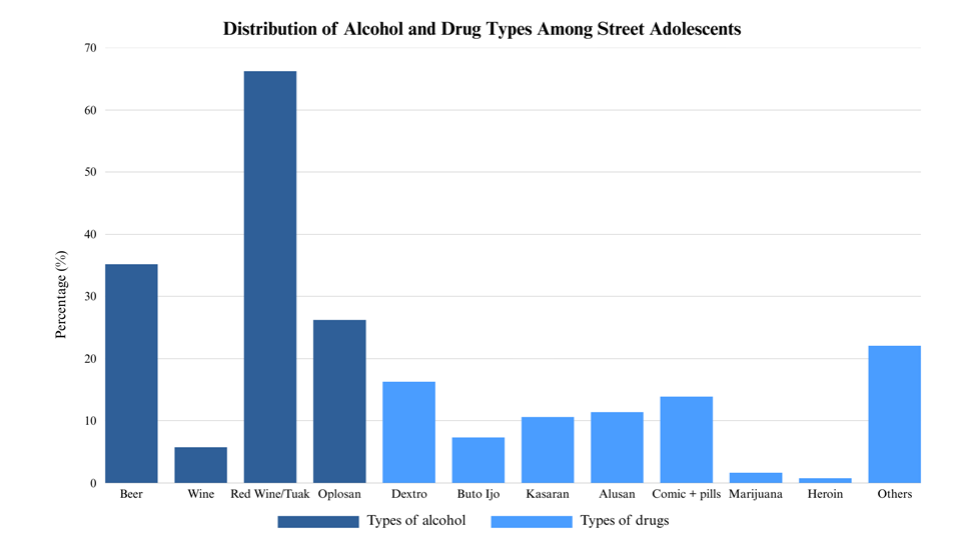
Distribution of alcohol and drug types among street adolescents. Link to the figure: https://www.canva.com/design/DAGqffuE91Y/vaFV7ZlrgMBdmrS2phh7SQ/edit?utm.

Table 1 shows several critical patterns in the behavioral profile of street adolescents. Specifically, 58.4% of respondents smoked 6-10 packs of cigarettes per week, suggesting habitual and possibly addictive patterns of tobacco use. Alcohol consumption was also prevalent among those who drank, with 66.3% reporting the use of traditional fermented beverages (tuak) and 26.2% consuming home-made alcohol *(oplosan).* This form was often associated with contamination and toxic effects due to unregulated production. Illegal drug use, although reported by a smaller subset (n = 57), showed complex and culturally specific dynamics. Local street terms such as *Buto Ijo, Kasaran,* and *Alusan*, which denoted varying perceived purity and psychoactive properties of methamphetamine, were commonly mentioned, suggesting a deep integration of drug culture within this population. More importantly, 22.1% reported the use of unidentified substances, and approximately 14% consumed cocktails of over-the-counter medications (*Comic* syrup and pills), indicating dangerous experimentation without medical supervision (Figure 2). Exposure to sexual content was approximately universal, with 62.5% of adolescents reporting frequent exposure to pornography mostly through mobile devices.

**Table 1:** Descriptive statistics of continuous variables among street adolescents in Central Java (n = 248)

**Variable**	**Mean**	**SD**
Age	14.69	2.823
Knowledge of sexuality	21.13	10.83
Attitudes towards sexuality	8.64	3.24
Friends’ sexual behavior	5.29	2.77

 Although 72.5% were identified as sexually abstinent, a significant proportion (27.5%) had initiated sexual activity, with 13.7% engaging in high-risk practices such as unprotected sex, transactional relationships, or multiple partnerships (Table 2). The youngest sexually active respondent was 11 years old, showing a profound public health concern regarding early sexual debut in the absence of protection and education. Additionally, peer influence appeared to play a major role, with 74.6% reporting that close friends had engaged in sexual activities, suggesting a strong normative pressure toward sexual risk-taking. A striking contrast was found between the high prevalence of permissive sexual attitudes (65.7%) and the low level of knowledge about sexual and reproductive health (only 21% adequate). This knowledge-attitude gap could contribute to misinformed decision-making and sustained engagement in unsafe behavior, particularly when reinforced by peer norms and media exposure.

**Table 2:** Frequency distribution of categorical variables among street adolescents in Central Java (n = 248)

**Variable**	**Number**	**Percent**
Gender		
Male	151	60.9
Female	97	39.1
Occupational status		
Beggars	84	33.9
Odd Job	59	23.8
No Job	105	42.3
Smoking behavior (packs/week)		
1-5	89	35.8
6-10	145	58.4
11-15	14	5.8
Drinking alcohol		
Ever	122	49.1
Never	126	50.9
Narcotics		
Ever	57	22.9
Never	191	77.1
Exposure to pornography (times/week)		
2-7	155	62.5
< 2	93	37.5
Sexual risk behavior		
Unsafe sex	32	13.7
Safe sex	35	13.3
Abstinence	180	72.5

###  Association Between Substance Use and Sexual Behavior

Table 3 shows key factors related to high-risk sexual behavior among street adolescents. Younger adolescents (10–14 years) were significantly more likely to engage in high-risk sex than the older population (*P* = 0.001), showing early vulnerability. Substance use showed the strongest associations, while narcotic users and drinkers had a tendency to report high-risk sex behavior (*P* = 0.001), emphasizing how disinhibition could increase sexual risk behavior. Highly permissive sexual attitudes and frequent exposure to pornography were also strongly related to high-risk behavior (*P* = 0.001), showing the influence of norms and media on adolescents’ decision-making.

**Table 3:** The association between substance use and sexual risk behavior in street adolescents (n = 248)

**Variables**	**High risk**		**Low risk**		***P *** **value**
	**Number**	**Percent**	**Number**	**Percent**	
Age (year)					0.001
10-14 (n = 154)	56	36.3	98	63.7	
15-18 (n = 94)	12	12.7	82	87.3	
Gender					0.906
Male (n = 151)	41	27.2	110	72.8	
Female (n = 97)	27	27.8	70	72.2	
Occupation					0.121
Beggars (n = 84)	32	38.1	52	61.9	
Odd Job (n = 59)	19	32.2	40	67.8	
No Job (n = 105)	17	16.2	88	67.8	
Smoking					0.001
Heavy smokers (n = 151)	60	39.7	91	60.3	
Light smokers (n = 97)	8	8.2	89	91.8	
Alcohol consumption					0.001
Drinks (n = 122)	57	46.7	65	53.3	
No (n = 126)	11	8.7	115	91.3	
Narcotics					0.001
Users (n = 57)	31	54.4	26	45.6	
No (n = 191)	37	19.4	154	80.6	
Knowledge of sexuality					0.466
Low (n = 34)	7	20.6	27	79.4	
Medium (n = 162)	44	17.7	118	72.8	
High (n = 52)	17	32.7	35	67.3	
Attitudes towards sexuality					0.001
Not permissive (n = 85)	7	8.2	78	91.8	
Intermediate (n = 129)	40	31	89	69	
Highly permissive (n = 34)	21	61.8	13	38.2	
Friends’ sexual behavior					0.001
Low risk (n = 63)	9	14.3	54	85.7	
High risk (n = 185)	59	31.8	126	85.7	
Exposure to pornography (times/week)					0.001
2-7 (n = 155)	62	40	93	60	
< 2 (n = 93)	6	6.5	87	93.5	

Table 4 presents the multivariate logistic regression analysis of factors associated with the sexual risk behavior of adolescents. In Model I, attitudes toward sexuality also played a substantial role. Compared to non-permissive attitudes, adolescents with highly permissive attitudes were even more likely to report sexual risk behavior (OR = 3.89, *P* = 0.001). This pattern suggested a dose-response relationship, where greater permissiveness was associated with higher behavior risk. Adolescents whose close friends showed high-risk sexual behavior (OR = 2.66, *P* = 0.028) were more likely to report risky sexual practices. This confirmed the significant role of peer norms in shaping adolescents’ decision-making.

**Table 4:** Results of multivariate logistic regression analysis (n = 248)

**Variables**	**Model I**		**Model II**		**Model III**	
	**Adjusted OR (95% CI)**	***P *** **value**	**Adjusted OR (95% CI)**	***P *** **value**	**Adjusted OR (95% CI)**	***P *** **value**
Age (year)						
10-14	1.00		-		-	
15-18	2.56 (1.08, 6.04)	0.030	-		-	
Attitudes towards sexuality						
Not permissive	1.00		-		-	
Highly permissive	3.89 (1.75, 8.63)	0.001	-		-	
Peers’ sexual behavior					-	
Low risk	1.00		-			
High risk	2.66 (1.13, 6.38)	0.028	-		-	
Exposure to pornography						
Seldom	1.00		1.00		-	
Often	3.17 (1.13, 8.86)	0.027	4.20 (1.57, 11.2)	0.004	-	
Alcoholic drinking						
Never	1.00		1.00		1.00	
Ever	2.07 (0.71, 5.97)	0.177	3.29 (1.25, 8.64)	0.016	4.38 (1.71, 11.2)	0.002
Smoking						
Light smoker	1.00		1.00		1.00	
Heavy smoker	1.84 (0.26, 2.68)	0.781	1.47 (0.50, 4.28)	0.475	2.17 (0.78, 6.00)	0.133
Narcotic use						
Never	1.00		1.00		1.00	
Ever	1.53 (0.69, 3.39)	0.292	1.95 (0.95, 4.01)	0.690	1.89 (0.93, 3.87)	0.780

 In Model II, frequent exposure to pornography was significantly associated with sexual risk behavior (OR = 4.20, *P* = 0.004), consistent with results from Model I (OR = 3.17, *P* = 0.027). Alcohol consumption remained a significant predictor in Model II (OR = 3.29, *P* = 0.016) and Model III (OR = 4.38, *P* = 0.002). This persistent association showed that alcohol use could be a robust risk behavior. In comparison, smoking (OR = 2.17, *P* = 0.133) and narcotic use (OR = 1.89, *P* = 0.78) did not show statistically significant associations in Model III. This suggested that substance could not independently predict sexual risk behavior when other factors are considered.

 To evaluate the model fit, the Hosmer and Lemeshow goodness-of-fit test was performed for all models. The results indicated no significant difference between the observed and expected frequencies in any of the models, suggesting a good fit to the data (Model I, χ^2^ = 6.25, df = 8, *P* = 0.62, Model II, χ^2^ = 8.03, df = 8, *P* = 0.43, and Model III, χ^2^ = 10.75, df = 8, *P* = 0.21). These non-significant *P* values (*P* > 0.05) suggest that the predicted probabilities from each model closely matched the observed outcomes, indicating an overall good fit.

## Discussion

 This study identified several factors associated with sexual risk behavior among street adolescents, including alcohol consumption, permissive sexual attitudes, peer sexual behavior, and frequent exposure to pornography. The results are in line with previous studies that have related substance use, particularly alcohol, with sexual risk behavior such as unprotected sex, multiple partners, and transactional sex,^33^ as well as early initiation and engagement with non-regular partners.^34^

 The multivariate analysis showed alcohol consumption as the only substance use factor significantly associated with high-risk sexual behavior across all models (OR = 3.26–4.38). This is consistent with previous studies showing that alcohol use posed a greater risk compared to marijuana.^35^ The psychoactive properties of alcohol, which impair cognitive function and lower inhibitions, contribute to impulsive sexual decision-making.^36^ In the context of Indonesian street adolescents, alcohol, which is often consumed as cheap and unregulated*oplosan, *is commonly used for recreation, emotional escape, and bonding. It is also associated with a party culture that normalizes impulsivity and risk.

 In comparison, smoking and narcotic use were not found to be statistically associated with sexual risk behavior in this study, diverging from some global literature.^18,37^ Smoking, particularly among male adolescents, was highly normalized and socially accepted, including marginalized groups like street adolescents, with prevalence rates of 85%–90%. Furthermore, smoking carries minimal social stigma and is widely perceived as a normative behavior, unrelated to sexual risk-taking. Narcotic use, which is serious and often reported in the vulnerable population, is significantly stigmatized in Indonesia, and it is associated with criminality, moral failure, and punitive legal consequences. Access to narcotics is limited, and usage is often hidden, leading to possible underreporting. When it does occur, there is a reflection of more complex psychosocial distress, such as depression or trauma, rather than a direct link to sexual behavior.

 Despite the vulnerability of street adolescents, 72.5% of respondents in this study reported abstaining from sexual activity, underscoring the strong influence of prevailing sociocultural norms of Indonesia. In Indonesian society, sexual relations are widely accepted only within the bounds of legal marriage, while premarital sex, particularly among adolescents, is stigmatized and morally condemned. These normative beliefs may not only shape behavior but also suppress truthful disclosure, particularly in studies comprising marginalized groups. Therefore, the high rate of reported abstinence may show both the internalization of dominant cultural expectations and the influence of shame, fear, or social desirability bias in self-reporting.

 Adolescents surrounded by peers with permissive sexual norms were more likely to engage in high-risk sexual behavior. This supported previous results that peer norms were powerful determinants of adolescents’ sexual behavior. Similarly, frequent exposure to pornography was associated with increased sexual risk. Other studies suggested that frequent consumption of sexual media could distort expectations about sex, reinforce objectification, and diminish self-regulation.^38^

 Although drugs and smoking were not statistically significant factors in this analysis, their widespread use remained concerning. Previous reports suggested that approximately 75% of street adolescents engaged in substance use,^39^ and misconceptions, such as believing that alcohol or drugs could prevent pregnancy.^18,37^ These results emphasized the need for targeted interventions.

 Globally, sexual risk behavior among street adolescents has been observed across regions such as Sub-Saharan Africa, Latin America, and Southeast Asia, though contextual drivers vary. In Sub-Saharan Africa, factors such as poverty, orphanhood, and housing instability contribute to early sexual debut and transactional sex. Meanwhile, in Latin America, gang exposure and displacement increase vulnerability. Contributing factors in parts of Southeast Asia include child labor and urban migration, which are associated with limited access to sexual health education. High-income countries, including Canada and the United Kingdom, have implemented integrated responses such as harm reduction outreach, adolescent-friendly clinics, and peer-based education. These services are often supported by strong institutional frameworks and multi-agency coordination. In comparison, Indonesia lacks comprehensive and coordinated interventions for street adolescents. Existing efforts remain fragmented and limited in scale. This underscores the need for culturally and structurally grounded strategies that draw from global models while addressing unique sociocultural realities of Indonesia.

 From a policy standpoint, these results suggest several directions. First, given the strong association of alcohol with sexual risk, prevention efforts should prioritize alcohol education, control policies, and harm-reduction initiatives among adolescents. Second, peer influence and permissive attitudes toward sex should be addressed through comprehensive age-appropriate sexuality education that includes critical thinking, boundary setting, and healthy relationships. Third, media literacy programs are also crucial in mitigating the influence of pornography. Fourth, accessible adolescent-friendly services should be offered through multisectoral collaborations comprising health providers, NGOs, and community leaders.^40^ Fifth, programs must be designed to meet the unique needs of street adolescents who are often neglected in mainstream public health interventions.

 Beyond these policy implications, future research should adopt longitudinal or experimental designs to capture the temporal dynamics of sexual risk behaviors in adolescents and to better establish causal relationships between alcohol use, peer influence, and sexual decision-making. Moreover, examining interaction effects would provide deeper insights into the mechanisms underlying these behaviors. These studies could also clarify whether protective factors (e.g., strong family communication or school engagement) moderate these relationships over time.

 The strengths of this study include the focus on an underrepresented and hard-to-reach group, as well as the use of multivariate analysis to control for confounders. However, this study has several limitations. First, the sample was geographically limited and primarily recruited from urban areas where NGOs are more active, which may not reflect the experiences of adolescents in rural or less resourced regions. Second, the use of purposive sampling, focusing on adolescents affiliated with NGOs, introduces potential selection bias and may overrepresent individuals with higher awareness of health and social services. Third, the study was conducted during the COVID-19 pandemic, which may have influenced adolescents’ behaviors and access to services. Lastly, the cross-sectional design hinders the ability to infer causality, limiting the conclusion on whether substance use preceded sexual risk behavior or vice versa.

**Highlights**

- Over a quarter of street adolescents reported high-risk sexual behavior.
- Alcohol use increased sexual risk with adjusted odds ratios up to 4.38.
- Frequent exposure to pornography was strongly linked to sexual risk behavior.
- Heavy smoking, alcohol use, and exposure to pornography were prevalent in this group.
- Findings highlight the urgent need for targeted interventions for street adolescents

## Conclusion

 In conclusion, this study showed that substance use was significantly associated with sexual risk behavior among street adolescents in Central Java. Factors such as age, peer attitudes and behaviors, parental attitudes, and perceptions of sexuality were identified as confounding variables. The association between substance misuse and sexual risk behavior showed the urgent need for targeted interventions that address both issues simultaneously. Adolescents engaged in high-risk behavior, particularly substance use and sexual risk-taking, required comprehensive and tailored treatment options to mitigate these risks. Interventions must be developed to address the specific needs of this vulnerable population, focusing on both substance abuse prevention and sexual health education. Public health efforts should prioritize the creation of adolescent-friendly services that integrate mental, physical, and social support, ensuring accessible and effective care for street adolescents. Additionally, future studies should aim to include a broader and more representative population and assess the long-term outcomes of interventions to deepen the understanding of critical public health concerns. This would enhance the capacity to design more effective strategies, reducing both substance use and sexual risk behavior.

## Acknowledgements

 The authors are grateful to the NGO, caregivers, and street adolescents who participated in this study. The authors are also grateful to Diponegoro University, Faculty of Public Health, for providing funds and enabling this research to run smoothly.

## Competing Interests

 The authors have no conflict of interests associated with the material presented in this study.

## Ethical Approval

 This study was reviewed and approved by the Ethics Committee of the Faculty of Public Health, Diponegoro University (192/EA/KEPK-FKM/2022).

## Funding

 This research received funding support from the Faculty of Public Health, Diponegoro University.
